# Combined exposure to fine particulate matter and high glucose aggravates endothelial damage by increasing inflammation and mitophagy: the involvement of vitamin D

**DOI:** 10.1186/s12989-022-00462-1

**Published:** 2022-03-29

**Authors:** Tsai-Chun Lai, Yu-Chen Chen, Hui-Hua Cheng, Tzu-Lin Lee, Jaw-Shiun Tsai, I.-Ta Lee, Kuo-Ti Peng, Chiang-Wen Lee, Lee-Fen Hsu, Yuh-Lien Chen

**Affiliations:** 1 Department of Anatomy and Cell Biology, College of Medicine, National Taiwan University, Taipei, 100233 Taiwan; 2 Department of Family Medicine, National Taiwan University Hospital, Taipei, 100225 Taiwan; 3 Center for Complementary and Integrated Medicine, National Taiwan University Hospital, Taipei, 100225 Taiwan; 4 School of Dentistry, College of Oral Medicine, Taipei Medical University, Taipei, 110301 Taiwan; 5 Department of Orthopaedic Surgery, Chang Gung Memorial Hospital, Puzi City, Chiayi County 613016 Taiwan; 6 College of Medicine, Chang Gung University, Guishan District, Taoyuan City, 333323 Taiwan; 7 Department of Nursing, Division of Basic Medical Sciences, and Chronic Diseases and Health Promotion Research Center, Chang Gung University of Science and Technology, Puzi City, Chiayi County 613016 Taiwan; 8 Department of Safety Health and Environmental Engineering, Ming Chi University of Technology, New Taipei City, 243303 Taiwan; 9 Department of Respiratory Care, Chang Gung University of Science and Technology, Puzi City, Chiayi County 613016 Taiwan; 10 Division of Neurosurgery, Department of Surgery, Chang Gung Memorial Hospital, Puzi City, Chiayi County 613016 Taiwan

**Keywords:** Particulate matter, Diabetes, Endothelial damage, ROS, Mitophagy, Inflammation, Vitamin D3

## Abstract

**Background:**

Cardiovascular diseases (CVDs) are related to particulate matter (PM_2.5_) exposure. Researchers have not clearly determined whether hyperglycemia, a hallmark of diabetes, exacerbates PM_2.5_-induced endothelial damage. Thus, this study aimed to investigate the combined effects of PM_2.5_ and high glucose on endothelial damage.

**Results:**

Here, we treated human umbilical vein endothelial cells (HUVECs) with 30 mM high glucose and 50 μg/mL PM (HG + PM) to simulate endothelial cells exposed to hyperglycemia and air pollution. First, we showed that HUVECs exposed to PM under high glucose conditions exhibited significant increases in cell damage and apoptosis compared with HUVECs exposed to PM or HG alone. In addition, PM significantly increased the production of reactive oxygen species (ROS) in HUVECs and mitochondria treated with HG and decreased the expression of superoxide dismutase 1 (SOD1), a free radical scavenging enzyme. The coexposure group exhibited significantly increased ROS production in cells and mitochondria, a lower mitochondrial membrane potential, and increased levels of the autophagy-related proteins p62, microtubule-associated protein 1 light chain 3β (LC3B), and mitophagy-related protein BCL2 interacting protein 3 (Bnip3). Moreover, autophagosome-like structures were observed in the HG + PM group using transmission electron microscopy. The expression of intercellular adhesion molecule-1 (ICAM-1) and vascular cell adhesion molecule-1 (VCAM-1) were also increased through the JNK/p38 signaling pathway in the HG + PM group. As a ROS scavenger, vitamin D treatment effectively protected cells under HG and PM conditions by increasing cell viability, reducing mitochondrial ROS production, and suppressing the formation of mitophagy and inflammation. Furthermore, diabetes was induced in mice by administering streptozotocin (STZ). Mice were treated with PM by intratracheal injection. Vitamin D effectively alleviated oxidative stress, mitophagy, and inflammation in the aortas of mice treated with STZ and PM.

**Conclusion:**

Taken together, simultaneous exposure to PM and high glucose exerts significant harmful effects on endothelial cells by inducing ROS production, mitophagy, and inflammation, while vitamin D reverses these effects.

**Supplementary Information:**

The online version contains supplementary material available at 10.1186/s12989-022-00462-1.

## Background

Diabetes is a metabolic disease characterized by hyperglycemia and insulin resistance that has been recognized as one of the main factors contributing to the global burden of disease and premature death [1]. At the same time, air pollution has been listed as a major threat to public health [2]. Multiple human studies have explored the potential link between exposure to ambient air pollutants and diabetes [3]. Diabetes increases vulnerability to particulate air pollution, which is related to impaired endothelial function [4]. Acute exposure to PM_2.5_ causes conduit artery vasoconstriction and increases blood pressure in healthy adults, and chronic PM_2.5_ exposure is associated with persistent endothelial dysfunction [5]. Because endothelial cells regulate blood pressure, atherosclerosis, arrhythmia, and thrombosis [6], endothelial dysfunction may be attributed to exposure to PM_2.5_, which may lead to an increased CVD risk and increased sensitivity of patients with diabetics to PM_2.5_ [7]. Therefore, further research is needed to evaluate the link between air pollution and diabetes, especially in terms of endothelial cell function.

Due to the high incidence of diabetes associated with air pollution, research on the mechanism underlying the epidemiological observation is very important for public health. The possible biological pathways might include oxidative stress, apoptosis, and autophagy in endothelial cells. PM- or diabetes-mediated toxicity has been linked to oxidative stress [8, 9], inflammation [10], and apoptosis [11]. Recently, interest in the role of autophagy in regulating various physiological processes has increased. Autophagy marker proteins such as LC3B and p62 are generally regarded as indicators of autophagy flux [12]. However, this regulatory mechanism is related not only to normal cell functions but also to the occurrence of autophagic cell death, which leads to the onset of various diseases, such as diabetes and inflammation [13]. In addition, mitophagy is a selective form of macroautophagy involving dysfunctional mitochondria or the selective degradation of excess mitochondria, which is particularly important for cardiovascular homeostasis and disease [14]. Some studies have shown that PM exposure induces liver fibrosis by triggering mitophagy [15], and hyperglycemia promotes the mitophagy of endothelial cells [16]. The two main pathways of mitophagy include the PTEN-induced hypothetical kinase (PINK1)-Parkin pathway and the Nip3-like protein X (NIX) and Bnip3 pathways [17]. However, little information is available about the potential mechanism of action of PM on autophagy and mitophagy in individuals with diabetes.

Vitamin D is a multifunctional steroid hormone that regulates calcium homeostasis, oxidative stress, inflammatory processes and cancer prevention [18]. An epidemiological study reported a novel association between lower 25-hydroxyvitamin D3 levels with a faster decrease in lung function and with a high risk of chronic obstructive pulmonary disease [19]. Recent studies indicate that vitamin D alleviates oxidative stress and inflammation induced by PM exposure [20] or hyperglycemia [21]. However, little information is available on the mechanism underlying the protective effect of vitamin D on ECs exposed to PM and hyperglycemia. In the present study, we showed that PM aggravated ROS production, mitophagy, and inflammation in high glucose-treated endothelial cells. Vitamin D attenuated high glucose- and PM-induced mitophagy and inflammation by inhibiting ROS accumulation.

## Results

### PM reduced cell viability and induced the apoptosis of endothelial cells treated with high glucose

**Fig. 1:**
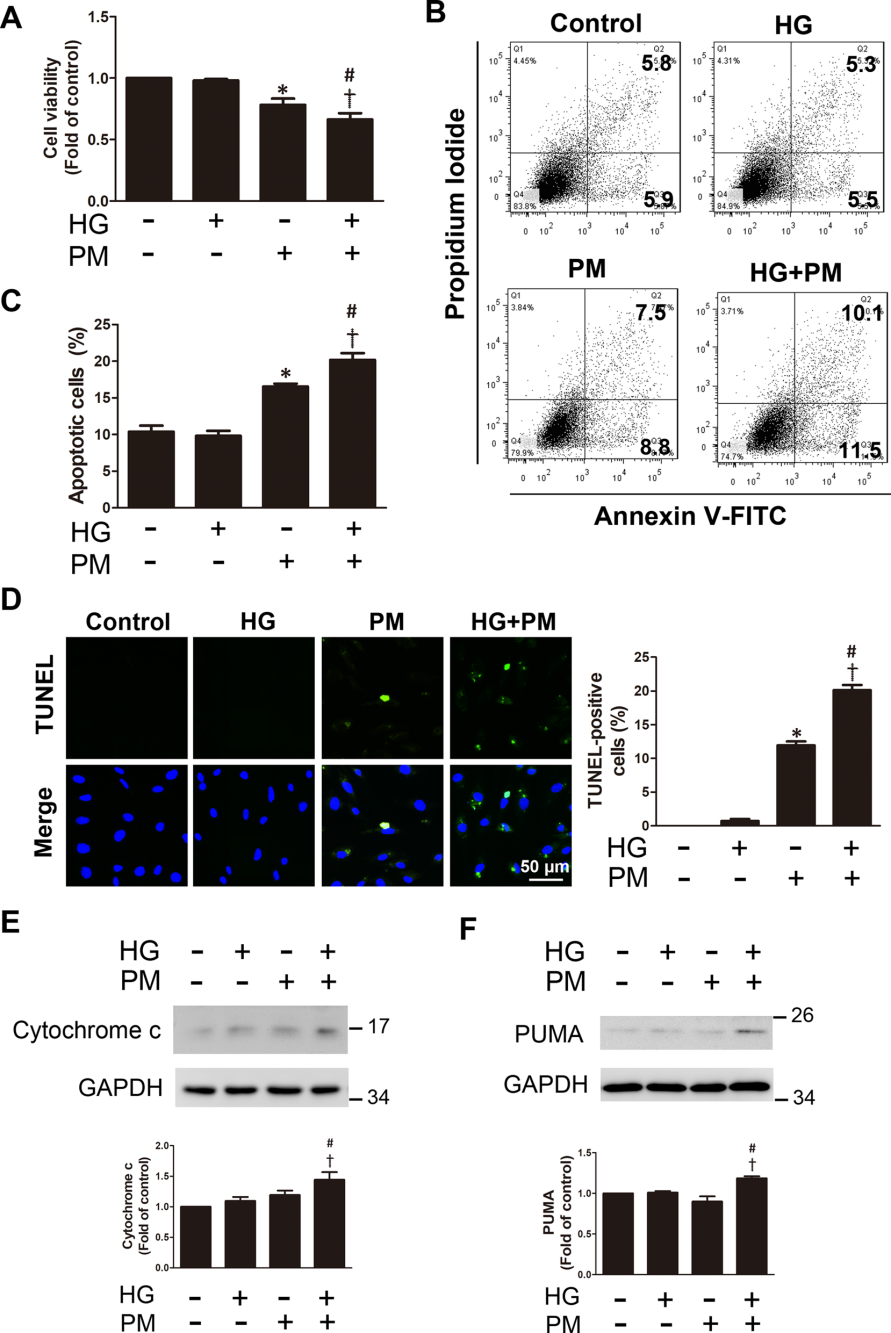
The effects of PM on the viability and apoptosis of endothelial cells treated with high glucose. HUVECs were pretreated with high glucose (30 mM) for 24 h and then treated with PM (50 μg/mL) for 8 h. **A** Cell viability was assessed using the MTT assay. **B, C** Cell death was detected using Annexin V-FITC/propidium iodide (PI) double staining. **D** Apoptosis was examined and quantified using the TUNEL assay (TUNEL-positive cells: green; nuclei: blue; bar = 50 μm). **E, F** The levels of cytochrome c and PUMA expression determined using Western blotting. *P < 0.05 compared with the control group; ^†^P < 0.05 compared with the HG group; ^#^P < 0.05 compared with the PM group

The administration of glucose at a concentration of up to 30 mM alone for 32 h did not cause significant cytotoxicity. PM treatment alone at a concentration of 50 μg/mL for 8 h significantly reduced cell viability compared with control cells. When the cells were pretreated with 30 mM glucose for 24 h and then treated with 50 μg/mL PM for 8 h (HG + PM), a significant decrease in cell viability was observed compared with the cells treated with high glucose or with PM alone (Fig. 1A). Here, cells were pretreated with 30 mM glucose for 24 h, treated with 50 μg/mL PM for 8 h, and then subjected to Annexin V-FITC/PI double staining to investigate the death of HUVECs under different conditions. PM increased both early (Q3) and late apoptotic cells (Q2) (Fig. 1B). The proportion of apoptotic cells (Q2 + Q3) was significantly increased in the HG + PM group compared with the high glucose or PM alone group (Fig. 1C). Consistent with these results, compared with high glucose or PM alone, the percentage of apoptotic cells was significantly increased in the HG + PM group, as measured using the TUNEL assay (Fig. 1D). We further examined the expression of apoptosis-related proteins, cytochrome c and PUMA in different groups. We found that the expression of these proteins was significantly increased after the HG + PM treatment (Fig. 1E, F). Our data showed that exposure to PM reduced cell viability, while exposure to PM and HG exacerbated endothelial cell death.

### PM aggravated ROS production in endothelial cells treated with high glucose, and 1,25(OH)_2_D_3_ pretreatment reduced this effect

**Fig. 2:**
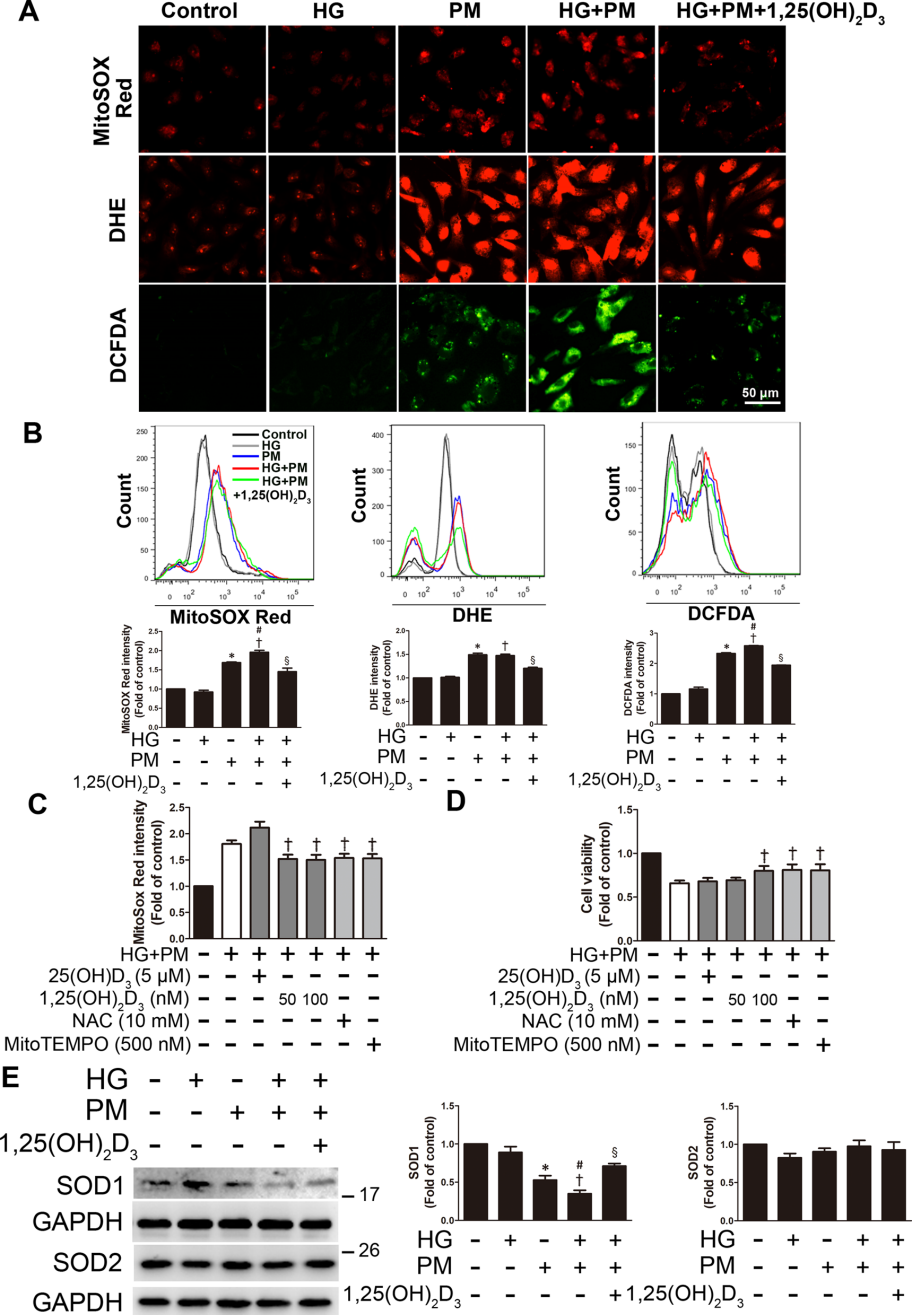
PM aggravated ROS production in endothelial cells treated with high glucose, and 1,25(OH)_2_D_3_ pretreatment attenuated this change. HUVECs were pretreated with high glucose (30 mM) for 24 h and then treated with PM (50 μg/mL) for 8 h. Before exposure to PM, HUVECs were pretreated with 100 nM 1,25(OH)_2_D_3_ for 12 h. **A** Mitochondrial and cellular ROS levels were detected by staining HUVECs with MitoSOX Red, DHE and DCFDA and examining them under a fluorescence microscope. Bar = 50 μm. **B** Mitochondrial and cellular ROS levels were detected by staining HUVECs with MitoSOX Red, DHE and DCFDA and analyzed using flow cytometry. **C** HUVECs were pretreated with 5 μM 25(OH)D_3_, 50 or 100 nM 1,25(OH)_2_D_3_, 500 nM MitoTEMPO and 10 mM NAC (an ROS scavenger) for 12 h and 1 h, respectively. Mitochondrial ROS was detected using MitoSOX Red and a flow cytometer. **D** HUVECs were pretreated with 5 μM 25(OH)D_3_, 50 or 100 nM 1,25(OH)_2_D_3_, 500 nM MitoTEMPO and 10 mM NAC for 12 h and 1 h, respectively. The MTT assay was used to evaluate cell viability. **E** The levels of SOD1 and SOD2 expression were detected using Western blotting. *P < 0.05 compared with the control group; ^†^P < 0.05 compared with the HG group; ^#^P < 0.05 compared with the PM group; ^§^P < 0.05 compared with the HG + PM group

Oxidative stress is one of the potential mechanisms of PM toxicity due to the presence of excess ROS [22]. MitoSOX Red, DHE and DCFDA were used to detect mitochondrial ROS, cellular superoxide anion and cytoplasmic H_2_O_2_ levels, respectively, in HUVECs under HG + PM conditions. PM exposure induced the production of mitochondrial ROS, cellular superoxide anion and cytoplasmic H_2_O_2 _according to fluorescence microscopy and flow cytometry, and the combined exposure to PM and HG exerted a synergistic effect on endothelial ROS production (Fig. 2A, B). Vitamin D was reported to be a very important antioxidant [21]. Two forms of vitamin D, 25(OH)D_3 _and 1,25(OH)_2_D_3_, were used in this study to determine the effects of vitamin D on HUVECs under high glucose- and PM-induced endothelial injury. In addition, NAC is an aminothiol and a synthetic precursor of cysteine ​​and GSH in cells; therefore, it is considered an antioxidant. Mito-TEMPO is an antioxidant for mitochondria that helps protect mitochondria from oxidative damage. We used these antioxidants to examine whether endothelial injury was caused by mitochondrial ROS production, as measured using the MitoSOX Red assay. ROS production was increased in the HG + PM group, while significantly reduced mitochondrial ROS levels were observed in HUVECs pretreated with 1,25(OH)_2_D_3 _(50 nM and 100 nM), Mito-TEMPO and NAC for 12 h and 1 h. However, HUVECs pretreated with 25(OH)D_3 _(5 μM) for 12 h did not exhibit reduced production of mitochondrial ROS (Fig. 2C). In addition, 1,25(OH)_2_D_3 _also significantly reduced HG + PM-induced cell superoxide anion and cytoplasmic H_2_O_2_ levels detected using DHE and DCFDA with fluorescence microscopy and flow cytometry (Fig. 2A, B). Next, we investigated the effects of vitamin D and antioxidants on cell viability following high glucose and PM treatment. 50 nM and 100 nM 1,25(OH)_2_D_3_ effectively decreased the MitoSOX Red intensity, while only 100 nM 1,25(OH)_2_D_3_ reversed the decrease in cell viability caused by high glucose and PM treatment (Fig. 2C, D). Therefore, a dose of 100 nM 1,25(OH)_2_D_3_ was considered sufficient to trigger the protective effect of vitamin D, and this dose was used in subsequent studies. NAC or Mito-TEMPO pretreatment also increased cell viability. However, HUVECs pretreated with 25(OH)D_3 _(5 μM) did not exhibit a change in cell viability (Fig. 2D). In addition, the expression of the ROS scavenger enzyme SOD1 was also reduced after high glucose and PM treatment, as shown in Western blots. However, cells pretreated with 100 nM 1,25(OH)_2_D_3 _displayed significantly increased SOD1 expression (Fig. 2E). Nevertheless, the expression of SOD2 remained constant after high glucose and PM treatment and even with 1,25(OH)_2_D_3_ pretreatment (Fig. 2E). Based on these results, exposure to PM or HG alone led to ROS production, and the combined effects of exposure to PM and HG accelerated ROS production in endothelial cells. These effects were reduced by the addition of 1,25(OH)_2_D_3_.

### PM aggravated mitochondrial injury in endothelial cells treated with high glucose, and 1,25(OH)_2_D_3_ pretreatment reduced this change

**Fig. 3:**
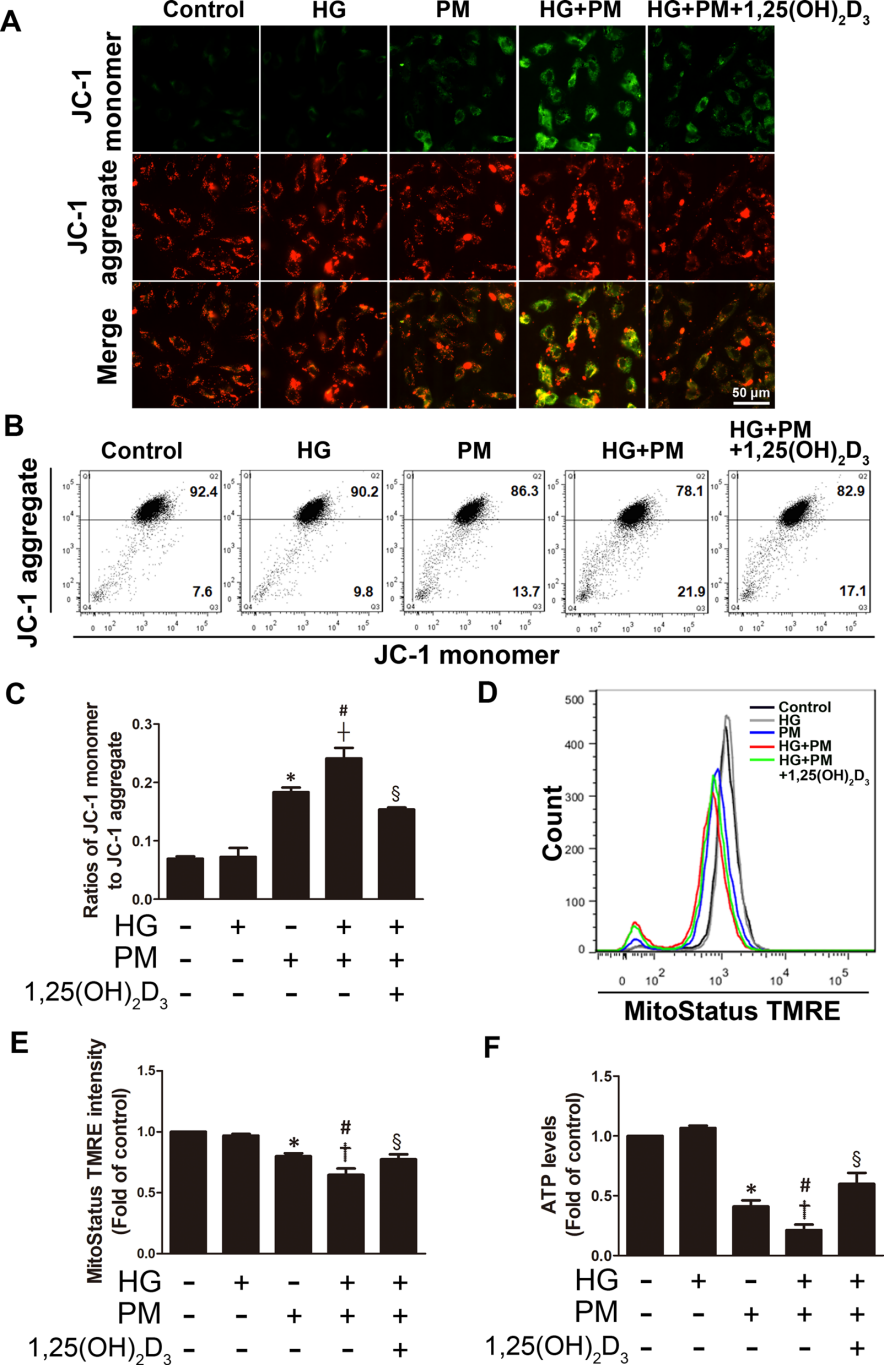
PM aggravated mitochondrial injury in endothelial cells treated with high glucose, and 1,25(OH)_2_D_3_ pretreatment reduced this change. HUVECs were pretreated with high glucose (30 mM) for 24 h and then treated with PM (50 μg/mL) for 8 h. HUVECs were pretreated with 100 nM 1,25(OH)_2_D_3_ for 12 h before PM exposure. **A** The JC-1 assay and fluorescence microscopy were used to examine the mitochondrial membrane potential. Bar = 50 μm. **B, C** The JC-1 assay and flow cytometry were used to examine mitochondrial membrane potential. Statistical analysis of the ratios of JC-1 monomer to JC-1 aggregates. **D, E** MitoStatus TMRE dye was used for the quantitative analysis of the mitochondrial membrane potential with flow cytometry. **F** The ATP assay was conducted to assess ATP levels. *P < 0.05 compared with the control group; ^†^P < 0.05 compared with the HG group; ^#^P < 0.05 compared with the PM group; ^§^P < 0.05 compared with the HG + PM group

Mitochondria are the powerhouses of every cell because they produce ATP supply as energy in the cell. Functional electron transport requires a complete electrochemical gradient, including the mitochondrial membrane potential (Δψm). Therefore, Δψm is essential for maintaining cell viability and is a key indicator of cell injury. Here, JC-1 and MitoStatus TMRE staining were used to examine Δψm. JC-1 is a cationic dye that forms aggregates in the presence of a high Δψm and emits red fluorescence, while remaining as monomers in the presence of a low Δψm and emitting green fluorescence. As shown in the results, the Δψm (increased green fluorescence) collapsed in both the PM alone and HG + PM groups, as determined using JC-1 staining and a fluorescence microscope (Fig. 3A). The results of JC-1 staining and subsequent flow cytometry were consistent with the fluorescence microscopy findings (Fig. 3B, C). PM exposure induced a decrease in the Δψm, and the combined exposure to PM and HG exerted a synergistic effect on decreasing the Δψm. However, 1,25(OH)_2_D_3 _treatment significantly reduced the effect (Fig. 3A–C). In addition, MitoStatus TMRE is a cationic lipophilic dye used to evaluate Δψm, as it accumulates in intact mitochondria. Δψm collapsed in both the PM alone and HG + PM groups based on MitoStatus TMRE staining, whereas 1,25(OH)_2_D_3 _treatment significantly increased Δψm compared to the HG + PM group (Fig. 3D, E). Moreover, ATP levels were evaluated by performing an ATP assay. The ATP levels in the PM alone and HG + PM groups were reduced, while 1,25(OH)_2_D_3_ treatment significantly reversed the change (Fig. 3F). Thus, high glucose and PM treatment caused significant mitochondrial damage, and 1,25(OH)_2_D_3_ pretreatment attenuated these effects.

### PM aggravated mitophagy in endothelial cells treated with high glucose, and 1,25(OH)_2_D_3_ pretreatment reduced this change

**Fig. 4:**
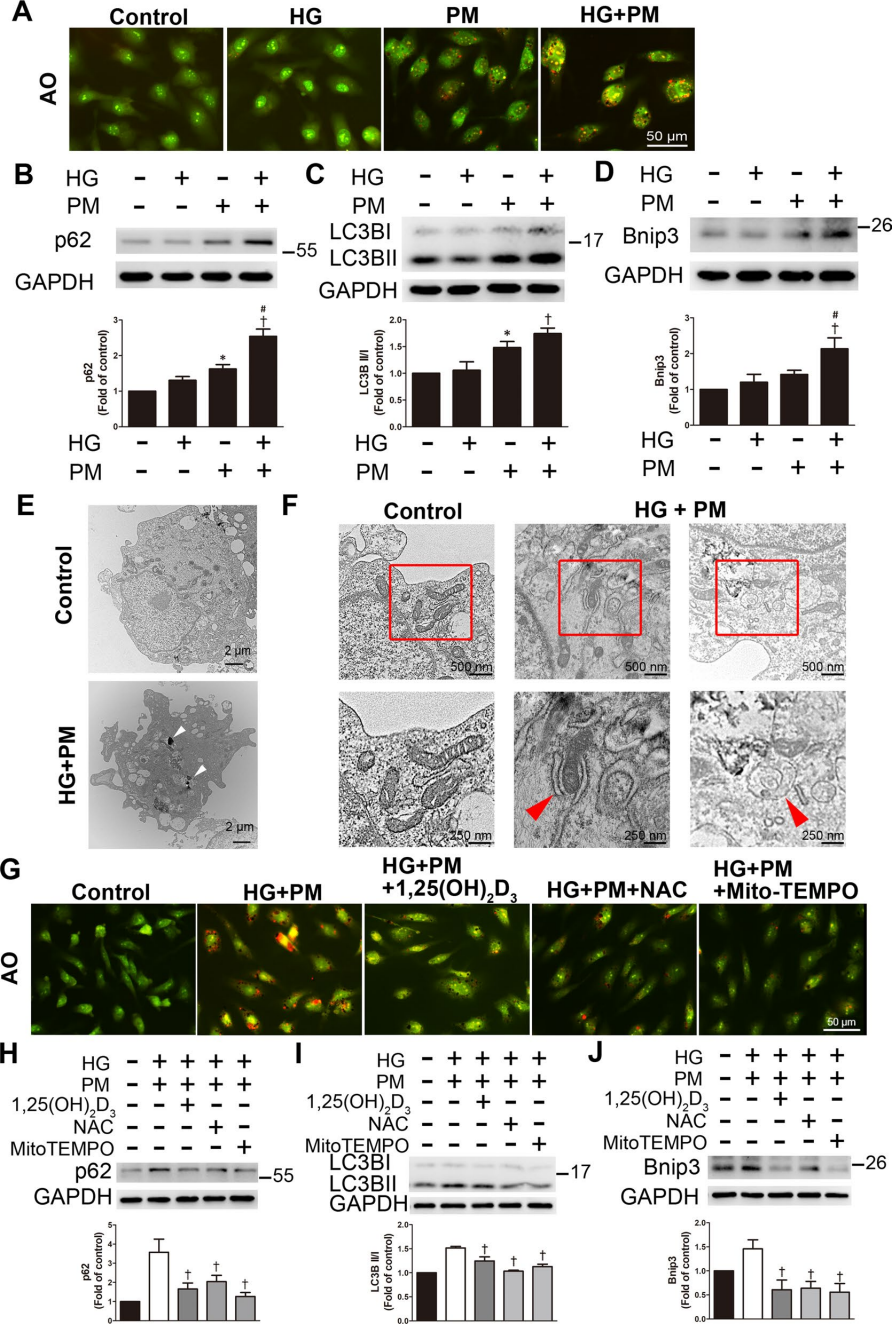
1,25(OH)_2_D_3_ reduced mitophagy induced by high glucose and PM exposure in HUVECs. HUVECs were pretreated with high glucose (30 mM) for 24 h and then treated with PM (50 μg/ml) for 8 h. **A** AO staining was used to detect the formation of autolysosomes and exhibited red fluorescence under a fluorescence microscope. Bar = 50 μm. **B–D** The levels of p62, LC3B, and Bnip3 expression were detected using Western blotting. **E, F** Transmission electron microscopy was used to detect the ultrastructure. White arrows indicated PM particles in the cytoplasm of HUVECs. Red arrows indicated the phagophore and the double-membrane autophagosome in the HG + PM group. Bars = 2 μm, 500 nm or 250 nm, as indicated in the panels. **G** HUVECs were pretreated with 100 nM 1,25(OH)_2_D_3_, 500 nM MitoTEMPO and 10 mM NAC for 12 h and 1 h before PM stimulation, respectively. AO was used to detect the autolysosomes. Bar = 50 μm. **H–J** HUVECs were pretreated with 100 nM 1,25(OH)_2_D_3_, 500 nM MitoTEMPO, and 10 mM NAC for 12 h and 1 h before PM stimulation, respectively. The levels of p62, LC3B and Bnip3 expression were assessed using Western blotting. *P < 0.05 compared with the control group; ^†^P < 0.05 compared with the HG group or HG + PM group; ^#^P < 0.05 compared with the PM group

Mitochondrial ROS production triggers mitochondrial damage, and then these damaged mitochondria are mainly engulfed and destroyed by mitophagy [14]. Here, AO staining was used to detect the formation of autolysosomes and is displayed as red fluorescence. Combined exposure to high glucose and PM treatment increased red fluorescence (Fig. 4A). PM exposure induced the expression of the mitophagy-related proteins p62 and LC3B, and the combined exposure to PM and HG exerted a synergistic effect on the levels of p62, LC3B, and Bnip3 (Fig. 4B–D). In addition, HG + PM treatment also increased the levels of p62 and LC3B in human aortic endothelial cells (HAECs) (Additional file 1: Fig. 1). Furthermore, we detected the morphology of HUVECs using transmission electron microscopy. We observed that the control endothelial cells retained the integrity of the cell membrane and mitochondria, with well-developed and clear cristae. HUVECs took up PM in the HG + PM group (Fig. 4E). At higher magnification, some swollen and ruptured mitochondria were observed in the HG + PM group. We also observed phagophore and double-membrane autophagosome structures in the HG + PM group (Fig. 4F). AO staining showed that 1,25(OH)_2_D_3 _(100 nM), NAC, and Mito-TEMPO suppressed red fluorescence, indicating that 1,25(OH)_2_D_3_ and antioxidants inhibited autophagy (Fig. 4G). 1,25(OH)_2_D_3,_ NAC, and Mito-TEMPO also reduced the increased levels of these three proteins induced by high glucose and PM treatment (Fig. 4h–j). These results suggested that high glucose and PM treatment increased mitophagy in HUVECs, while 1,25(OH)_2_D_3_ reduced these changes.

### PM aggravated inflammation of endothelial cells treated with high glucose, and 1,25(OH)_2_D_3_ pretreatment reduced inflammation

**Fig. 5:**
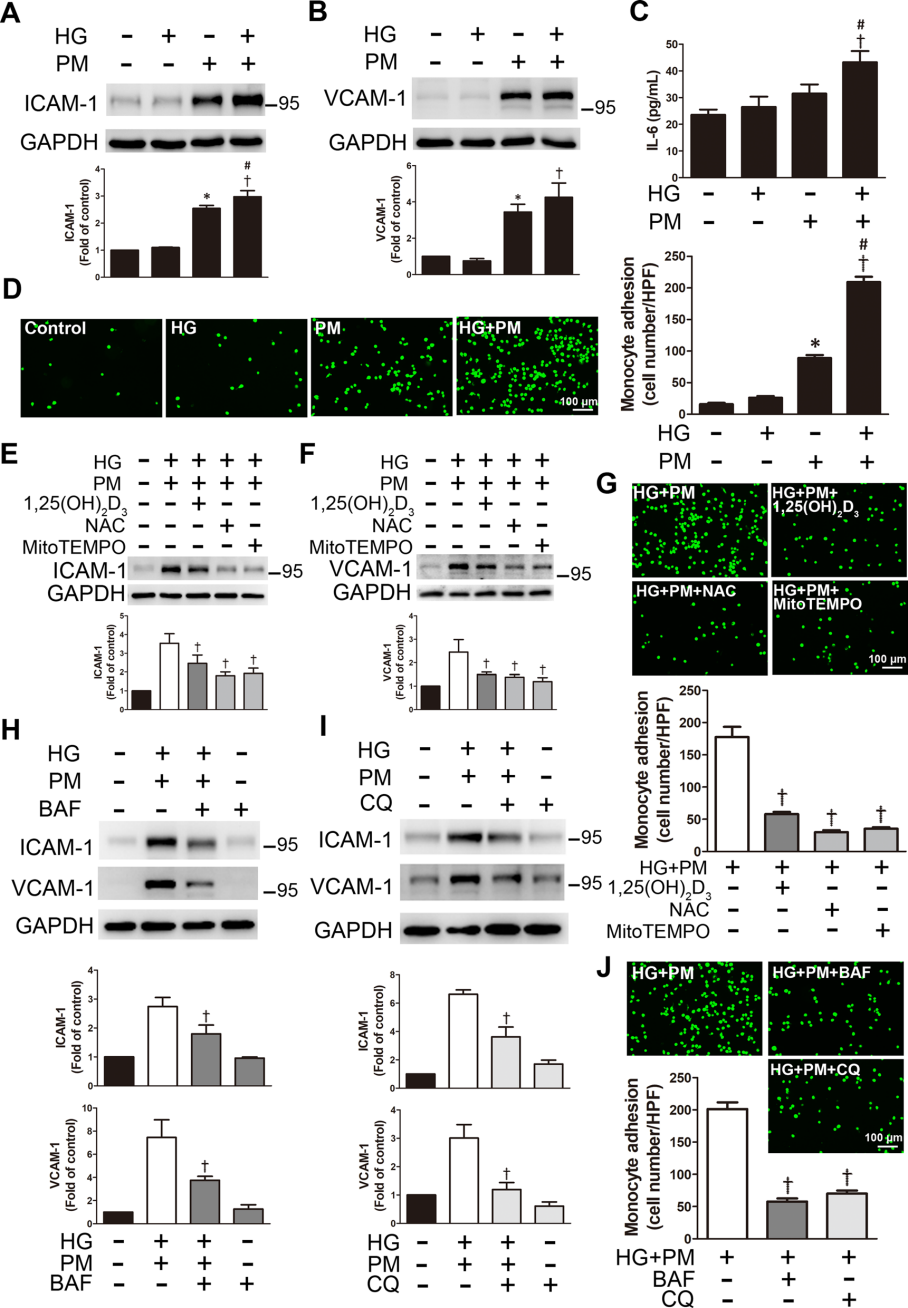

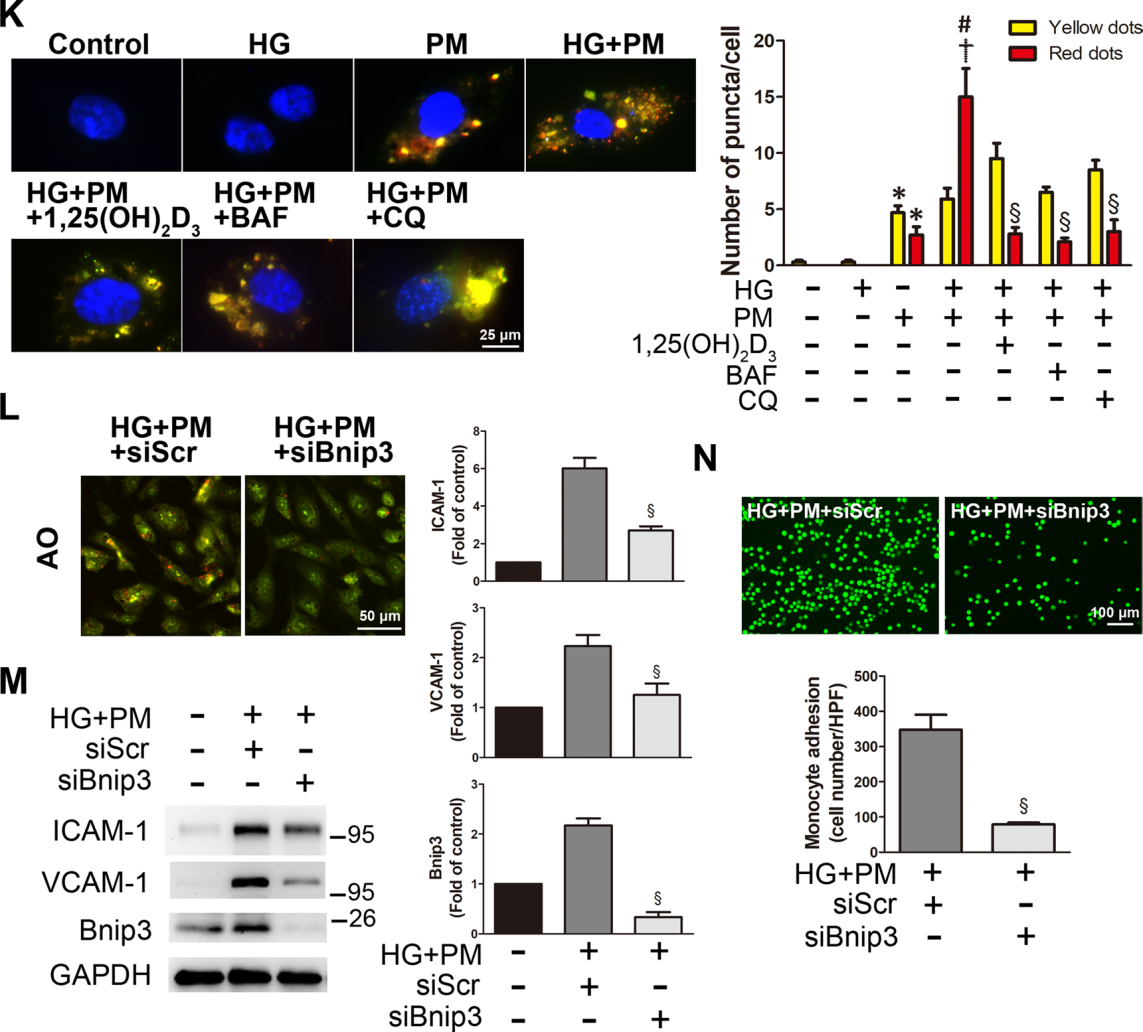
1,25(OH)_2_D_3_ alleviated endothelial inflammation in high glucose and PM-treated HUVECs. HUVECs were pretreated with high glucose (30 mM) for 24 h and then treated with PM (50 μg/ml) for 8 h. **A, B** The expression of ICAM-1 and VCAM-1 was detected using Western blotting. **C** The levels of IL-6 were measured using ELISA. **d** Representative fluorescence images showing the adhesion of fluorescein-labeled THP-1 cells to HUVECs. **E, F** HUVECs were pretreated with 100 nM 1,25(OH)_2_D_3_, 500 nM MitoTEMPO and 10 mM NAC for 12 h and 1 h, respectively. The expression of ICAM-1 and VCAM-1 was detected using Western blotting. **G** HUVECs were pretreated with 100 nM 1,25(OH)_2_D_3_, 500 nM MitoTEMPO and 10 mM NAC for 12 h and 1 h, respectively. Representative fluorescence images showing fluorescein-labeled THP-1 cells adhering to HUVECs. **H, I** HUVECs were pretreated with 100 nM BAF or 10 μM CQ for 1 h before PM exposure. The expression of ICAM-1 and VCAM-1 was detected using Western blotting. **J** HUVECs were pretreated with 100 nM BAF or 10 μM CQ for 1 h before PM exposure. Representative fluorescence images showing fluorescein-labeled THP-1 cells adhering to HUVECs. **K** Autophagy flux analysis with tandem sensor RFP-GFP-LC3B. HUVECs received the aforementioned treatment. Cells were stained with DAPI. The autophagosomes and autolysosomes were shown as the yellow dots and red dots, respectively. Bar = 25 μm. The number of autophagosomes and autolysosomes were counted per cell. **L** HUVECs were transfected with a scrambled siRNA (siScr) or siRNA targeting Bnip3 (siBnip3) and then exposed to high glucose and PM. AO was used to detect the formation of autolysosomes. Bar = 50 μm. **M** HUVECs were transfected with a scrambled siRNA (siScr) or siRNA targeting Bnip3 (siBnip3), followed by high glucose and PM exposure. The expression of ICAM-1, VCAM-1 and Bnip3 was detected using Western blotting. **N** HUVECs were transfected with a scrambled siRNA (siScr) or siRNA targeting Bnip3 (siBnip3), and then exposed to high glucose and PM. Representative fluorescence images showing the adhesion of fluorescein-labeled THP-1 cells to HUVECs. *P < 0.05 compared with the control group; ^†^P < 0.05 compared with the HG group or HG + PM group; ^#^P < 0.05 compared with the PM group; ^§^P < 0.05 compared with the HG + PM group or HG + PM + siScr group

When ROS are continuously produced, this condition usually leads to chronic inflammation and endothelial damage and is involved in the pathophysiology of cardiovascular diseases [23]. PM from air pollution is associated with endothelial damage, systemic inflammation, oxidative stress, and the progression of atherosclerosis [24]. The expression of ICAM-1 and VCAM-1 was examined using Western blotting to examine the effects of high glucose and PM on inflammation. The expression of these proteins was significantly increased following high glucose and PM treatment (Fig. 5A, B). Combined exposure to high glucose and PM also increased the level of ICAM-1 in HAECs (Additional file 1: Fig. 1). In addition, we also measured the level of the inflammatory cytokine IL-6 using ELISA. Compared with the HG alone group or the PM alone group, the level of IL-6 in the supernatant of the HG + PM group was increased, suggesting that high glucose and PM exposure induced inflammation and the secretion of proinflammatory cytokines (Fig. 5C). Since the cell adhesion molecules ICAM-1 and VCAM-1 are essential for monocyte adhesion, the effects of high glucose and PM on monocyte adhesion were examined. PM exposure significantly increased the adhesion of monocytes to HUVECs. When HUVECs were treated with HG + PM, the binding of monocytes was significantly enhanced (Fig. 5D). We also evaluated the effects of 1,25(OH)_2_D_3 _on inflammation in endothelial cells exposed to high glucose and PM. Notably, 1,25(OH)_2_D_3 _(100 nM)_, _NAC, and Mito-TEMPO decreased the expression of ICAM-1 and VCAM-1 following high glucose and PM treatment (Fig. 5E, F). Additionally, 1,25(OH)_2_D_3 _(100 nM)_, _NAC, and Mito-TEMPO decreased monocyte adhesion to endothelial cells induced by high glucose and PM treatment (Fig. 5G). Autophagy flux in endothelial cells exposed to high glucose and PM was further studied by applying two autophagy inhibitors, chloroquine (CQ) and bafilomycin A1 (BAF), which induce the accumulation of the LC3B-II protein by blocking the fusion between autophagosomes and lysosomes or suppressing the acidification of the lysosomes [25, 26]. After pretreatment with BAF (100 nM) or CQ (10 μM), the expression of ICAM and VCAM-1 was significantly decreased in the HG + PM group (Fig. 5H, I), indicating that autophagy induced the expression of ICAM-1 and VCAM-1 in endothelial cells. Cells treated with BAF or CQ also exhibited significantly reduced monocyte adhesion induced by HG + PM (Fig. 5J). In addition, Autophagy Tandem Sensor RFP-GFP-LC3B assay was used to evaluate the autophagy flux in HUVECs. HUVECs treated with PM induced the formation of autophagosomes (yellow dots) and autolysosomes (red dots), and red dots significantly increased in the HG + PM group. However, 1,25(OH)_2_D_3_, BAF and CQ pretreatment reduced the formation of autolysosomes (Fig. 5K). Moreover, transfection with the siRNA targeting Bnip3 prevented mitophagy. HUVECs were transfected with siRNA targeting Bnip3 and then treated with HG + PM, and the red fluorescence emitted by AO staining was reduced (Fig. 5L). Bnip3 knockdown significantly decreased the upregulation of ICAM-1 and VCAM-1 in HUVECs treated with HG + PM and substantially mitigated the adhesion of monocytes to HUVECs (Fig. 5M, N). Taken together, PM increased cell damage by inducing ROS production, mitophagy and inflammation in a high glucose environment, and 1,25(OH)_2_D_3_ protected HUVECs by eliminating oxidative stress and suppressing mitophagy. In addition, impeding mitophagy by Bnip3 knockdown also prevented the subsequent inflammation of HUVECs.

### Combined exposure to PM and HG induced inflammation and mitophagy through the JNK and p38 signaling pathways

**Fig. 6:**
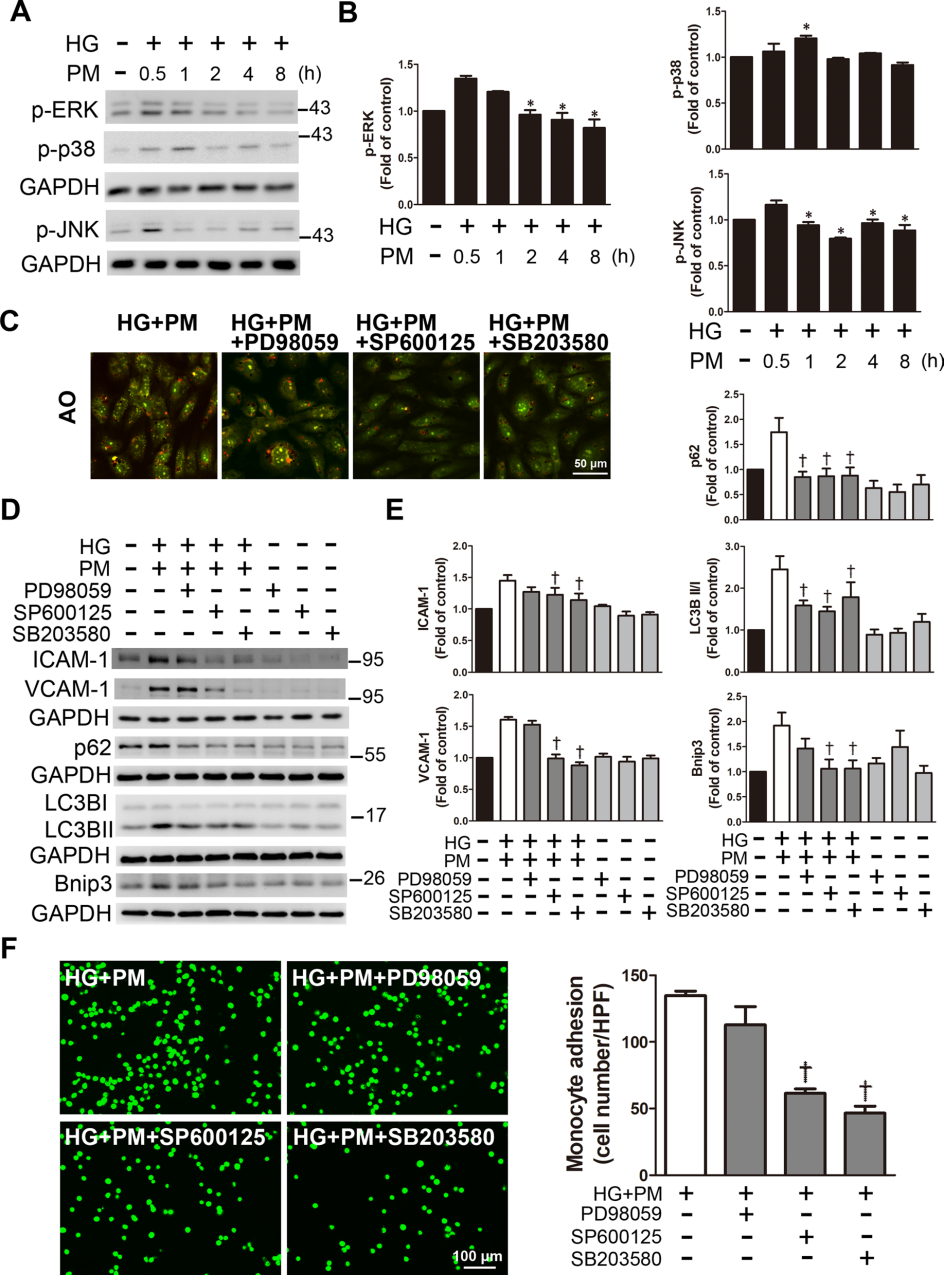
Mitophagy and inflammation induced by high glucose and PM in HUVECs were regulated by the JNK and p38 signaling pathways. HUVECs were pretreated with high glucose (30 mM) for 24 h and then treated with PM (50 μg/ml) for 8 h or a specified period. **A, B** The levels of p-ERK, p-p38, and p-JNK were detected at different time points (0, 0.5, 1, 2, 4, and 8 h) using Western blotting. **C** HUVECs were pretreated with PD98059 (10 μM), SP600125 (5 μM) or SB203580 (15 μM) for 1 h before PM stimulation. AO was used to detect the formation of autolysosomes. Bar = 50 μm. **D, E** HUVECs were pretreated with PD98059 (10 μM), SP600125 (5 μM) or SB203580 (15 μM) for 1 h before PM stimulation. The expression levels of ICAM-1, VCAM-1 p62, LC3B and Bnip3 were detected using Western blotting. **F** HUVECs were pretreated with PD98059 (10 μM), SP600125 (5 μM) or SB203580 (15 μM) for 1 h before PM stimulation. Representative fluorescence images showing the adhesion of fluorescein-labeled THP-1 cells to HUVECs. *P < 0.05 compared with the HG + PM 0.5 h group; ^†^P < 0.05 compared with the HG + PM group

We examined whether high glucose and PM activated the MAPK signaling pathway. Here, HUVECs were treated with PM for the indicated durations (0, 0.5, 1. 2, 4, or 8 h) in a high glucose environment. The levels of p-ERK, p-JNK, and p-p38 increased after treatment with PM for 0.5 h and 1 h in a high-glucose environment, and then significantly decreased (Fig. 6A, B). Then, before exposure to PM, HUVECs were pretreated with PD98059 (ERK inhibitor), SP600125 (JNK inhibitor) or SB203580 (p38 inhibitor) for 1 h. The assessment of autolysosomes by AO staining showed that HUVECs pretreated with PD98059 emitted red fluorescence, similar to the HG + PM group, while pretreatment with SP600125 and SB203580 reduced the red fluorescence (Fig. 6C). Next, pretreatment with SP600125 and SB203580 decreased the expression levels of ICAM-1, VCAM-1 and Bnip3, while pretreatment with PD98059, SP600125 and SB203580 significantly decreased the expression levels of p62 and LC3B (Fig. 6D, E). SP600125 and SB203580 significantly reduced the adhesion of monocytes treated with HG + PM (Fig. 6F). Based on these results, high glucose and PM induced inflammation and mitophagy through the phosphorylation of JNK and p38.

### 1,25(OH)_2_D_3_ reduced ROS production, inflammation, and mitophagy in the aortas of diabetic mice exposed to PM

**Fig. 7:**
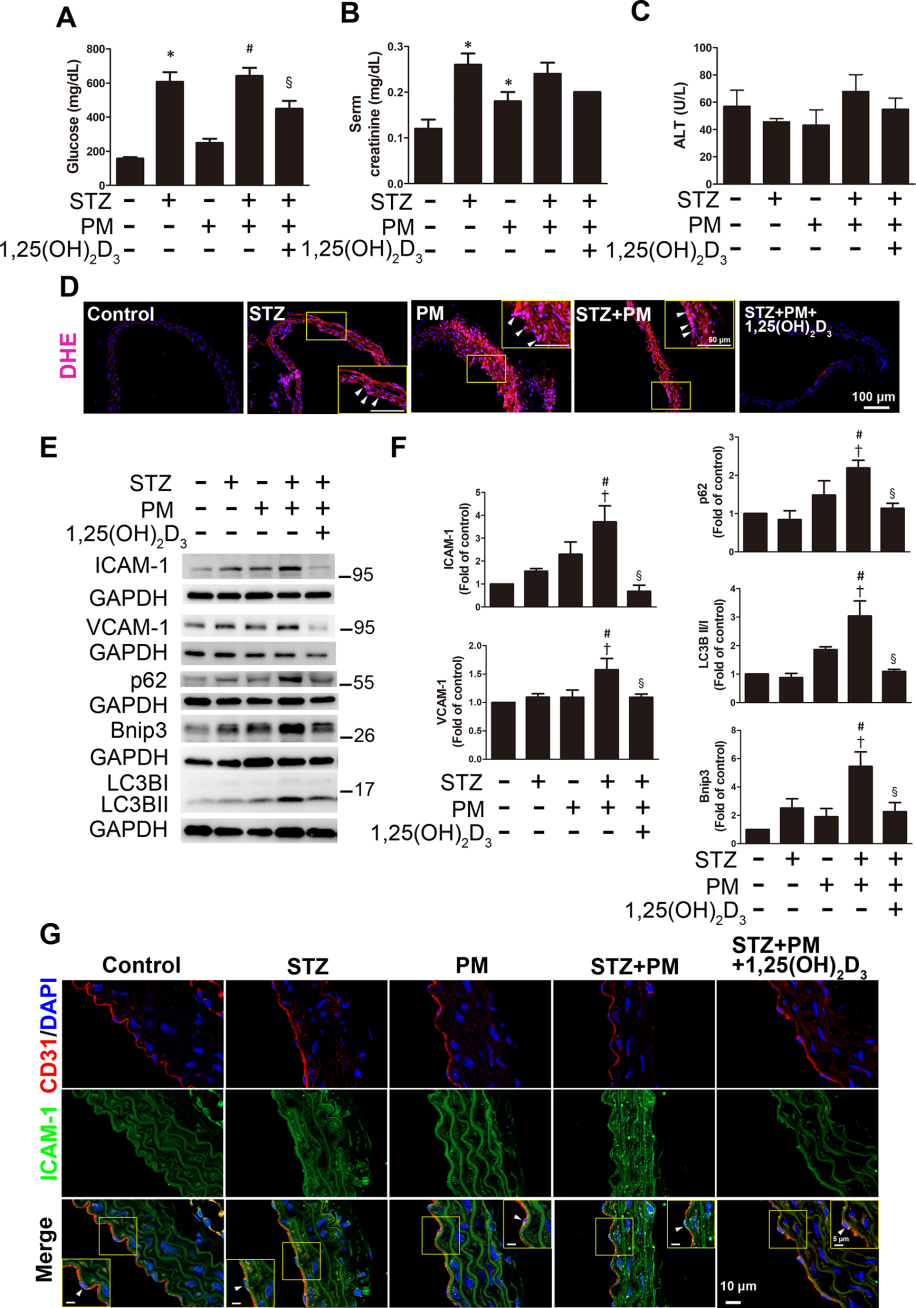
The effects of 1,25(OH)_2_D_3_ on mouse aortas under high glucose and PM conditions. Diabetes was induced in mice by intraperitoneally injecting STZ (55 mg/kg). Mice received PM (10 mg/kg) via intratracheal injection under anesthesia to simulate exposure to air pollution. 1,25(OH)_2_D_3_ was administered at a dose of 7 μg/kg per day for two weeks. The levels of **A** blood glucose, **B** serum creatinine and **C** ALT were analyzed. **D** DHE staining showed the level of oxidative stress in the mouse aorta. Red fluorescence in sections incubated with DHE indicated O_2_^•−^ production; blue fluorescence in sections stained with DAPI indicated the cell nucleus. Endothelial cells are indicated with white arrowheads. Bar = 100 μm. **E, F** The expression levels of ICAM-1, VCAM-1, p62, LC3B, and Bnip3 were detected using Western blotting. **G** The colocalization analysis of immunohistochemical staining showed that the expression of ICAM-1 in endothelial cells (positive for CD31) in the STZ + PM group was stronger than that in control mice. Yellow boxes show the magnification of endothelial cells stained with ICAM-1. Red fluorescence in sections indicated CD31; green fluorescence in sections indicated ICAM-1; blue fluorescence in sections indicated the cell nucleus. White arrowheads indicated the endothelial cells. Bars = 5 or 10 μm as indicated in the panels. *P < 0.05 compared with the control group; ^†^P < 0.05 compared with the STZ group; ^#^P < 0.05 compared with the PM group; ^§^P < 0.05 compared with the STZ + PM group

Diabetes was induced in mice by intraperitoneally injecting STZ (55 mg/kg), and PM (10 mg/kg) was injected into the trachea to simulate the air pollution environment. Vitamin D (1,25(OH)_2_D_3_) was injected intraperitoneally at a dose of 7 μg/kg per day for two weeks to examine its effect on diabetic mice exposed to PM. Then, blood glucose, serum creatinine, and alanine aminotransferase (ALT) levels were measured at the end of the experiment. Compared with the STZ + PM group, 1,25(OH)_2_D_3_ significantly reduced the blood glucose levels in STZ-induced diabetic mice with PM exposure (STZ + PM + 1,25(OH)_2_D_3_), indicating that vitamin D mitigated hyperglycemia in diabetic mice. (Fig. 7A). In addition, renal function was evaluated by measuring the serum creatinine level. The creatinine level in diabetic mice (STZ) was significantly higher than that in control mice; however, no significant difference was observed between the STZ + PM group and the STZ + PM + 1,25(OH)_2_D_3_ group (Fig. 7B). Furthermore, no significant difference in ALT levels (liver function index) was observed among these groups (Fig. 7C).

We verified whether 1,25(OH)_2_D_3_ affected ROS production in the aortas of diabetic mice exposed to PM using DHE was used to detect the superoxide anion (O_2_^•−^) levels. Diabetic mice (STZ), PM-exposed group (PM), and PM-exposed diabetic mice (STZ + PM) induced the production of O_2_^•−^, as indicated by the red fluorescence. Following 1,25(OH)_2_D_3_ treatment, the red fluorescence was reduced (Fig. 7D), indicating that 1,25(OH)_2_D_3_ inhibited O_2_^•−^ production in the mouse aorta.

We also examined the effects of 1,25(OH)_2_D_3_ on inflammation and mitophagy in STZ-induced diabetic mice exposed to PM using Western blotting. The expression of ICAM-1, VCAM-1, p62, Bnip3 and LC3B was increased in the STZ + PM group. However, 1,25(OH)_2_D_3_ treatment reduced the levels of these proteins (Fig. 7E, F), indicating that 1,25(OH)_2_D_3_ alleviated inflammation and mitophagy in the aortas of PM-treated diabetic mice. The colocalization analysis of immunohistochemical staining showed that the expression of ICAM-1 in endothelial cells (positive for CD31) in the STZ + PM group was stronger than that in the control group (Fig. 7G). Taken together, these findings suggested that 1,25(OH)_2_D_3_ protected the aortas of diabetic mice exposed to PM by reducing ROS production, inflammation, and mitophagy.

## Discussion

The main findings of this study indicated that exposure to PM in the HG milieu exacerbated apoptosis and inflammation by inducing endothelial oxidative stress and mitophagy. The underlying signaling pathway included p38 and JNK. In addition, vitamin D treatment reduced the harmful effects of combined exposure to PM and HG. Furthermore, intratracheal injection of PM also increased oxidative stress, inflammation and mitophagy in STZ-induced hyperglycemic mice, and vitamin D alleviated this stimulation. Vitamin D exerts antioxidant effects to ameliorate the injury caused by air pollution in a high glucose environment.

The link between air pollution or diabetes mellitus and the progression of cardiovascular diseases has become a prevalent issue in the past few decades [27]. Previous studies have shown that endothelial injury-associated apoptosis plays a crucial role in high glucose- or PM-induced disease progression [28, 29]. As part of our examination of EC viability and apoptosis, exposure to 50 μg/mL PM for 8 h significantly reduced cell viability and increased apoptosis, while exposure to 30 mM HG (i.e., simulating the diabetic environment) for 32 h did not. The addition of 30 mM glucose to culture medium is generally considered a standard diabetes-like medium [30]. Consistent with our findings, ECs treated with 30 mM HG for 24–48 h did not show a change in viability [29]. In contrast, after exposure to 33 mM glucose for 36 to 48 h, high glucose increased the number of apoptotic ECs [31]. Another report also showed that HUVECs treated with 33 mM glucose for 72 h underwent apoptosis [32]. We demonstrated that exposure to PM and 30 mM glucose significantly reduced cell viability when compared with the combined exposure to PM and low doses of glucose (Additional file 1: Fig. 2). Importantly, our current study revealed that PM treatment under high glucose conditions aggravated cell apoptosis. Furthermore, ROS generation is a key mechanism of HG- or PM-induced diseases [8, 28]. Both cellular and mitochondrial ROS levels were significantly increased following high glucose and PM treatment in the present study. Additionally, in this experiment, simultaneous exposure to PM and HG reduced the expression of SOD1, an antioxidant enzyme [33], while another antioxidant enzyme SOD2 remained unchanged. Mitochondria are the main ROS generators via electron leakage to molecular oxygen under pathological conditions [34]. In our study, PM decreased the mitochondrial membrane potential and ATP concentration in the HG environment, which was induced by ROS generation. The present study shows that exposure to PM and HG exacerbates endothelial damage. The underlying mechanisms include increased mitochondrial oxidative stress and subsequent mitochondrial dysfunction.

Autophagy plays a vital role in cell apoptosis and may further impair endothelial function [35]. We next explored whether PM affects the autophagy response of ECs under high glucose conditions. Autophagy, which is characterized by the degradation of large numbers of damaged organelles, can be monitored by detecting the key indicators of autophagy flux, such as LC3B-II and p62 [12]. Furthermore, Bnip3 is associated with mitophagy, which is a specialized type of autophagy that targets mitochondria for elimination and cell death [36]. According to recent studies, autophagy is involved in EC damage caused by hyperglycemia [16]. In addition, PM exposure triggers autophagy in several cell lines, including HUVECs and mouse aortic endothelial cells [11, 37]. In the present study, PM significantly induced autophagy in an HG environment by increasing the levels of the autophagy flux indicators LC3B-II and p62 and the mitophagy-related protein Bnip3. AO staining and TEM showed that exposure to PM and HG also significantly increased the number of autophagosomes. Previous reports indicate that autophagy is a double-edged sword that may elicit protective or destructive functions. Therefore, autophagy can protect cells, but it may also cause cell damage [38]. We found that the increased levels of apoptosis, ROS production and inflammation were closely associated with the increases in autophagy and mitophagy under high glucose and PM conditions, as evidenced by the increased expression levels of p62, LC3B and Bnip3. Therefore, we proved that high glucose and PM-induced autophagy and mitophagy exacerbated EC damage in the present study.

EC injury is closely related to inflammation [39]. As shown in the present study, PM exposure in an HG environment significantly increased the expression of ICAM-1 and VCAM-1. In addition, the combination of a high glucose pretreatment and PM exposure increased the level of the inflammatory cytokine IL-6, which is similar to a previous study showing that PM alone induced inflammation through systemic IL-6 activation [40]. Importantly, our data showed that coexposure to PM and HG aggravated inflammation compared to exposure to a single risk factor (PM or HG). Previous reports have revealed that inflammation and environmental stress are associated with the phosphorylation of proteins in the MAPK pathway [41], especially the JNK and p38 proteins [42]. Consistent with our research, SP600125 (JNK inhibitor) and SB203580 (p38 inhibitor), but not PD98059 (ERK inhibitor), significantly eliminated the increased levels of ICAM-1 and VCAM-1 after high glucose and PM treatment. In addition, PD98059, SP600125 and SB203580 reduced the levels of the indices of autophagy flux p62 and LC3B, while SP600125 and SB203580 reduced the level of the mitophagy-associated protein Bnip3. Based on these data, the JNK and p38 signaling pathways are involved in inflammatory responses and mitophagy following high glucose treatment and PM exposure.

Little is known about the effect of vitamin D treatment on endothelial damage in individuals with diabetes living in highly polluted urban areas. In addition, there is still controversy as to whether vitamin D supplementation has a protective effect on CVD. Numerous meta-analyses did not observe an association between vitamin D and the protection of CVD [43, 44]. Nevertheless, vitamin D deficiency continues to attract considerable attention because of claims that a proper status may reduce the risk of a wide range of diseases [45]. Recent studies have reported close relationships between vitamin D and cardiovascular diseases, diabetes, cancer, autoimmune diseases, inflammation and chronic diseases [46]. Vitamin D deficiency or insufficiency potentially increases the risk of CVD by activating the inflammatory response, which may lead to increased arterial stiffness and endothelial dysfunction [47]. Vitamin D improves endothelial function and glucose homeostasis and reduces oxidative stress and inflammation [48]. Vitamin D exerts a beneficial effect on the β-cell function and normal immunity of patients with diabetes [49]. Another study also investigated the antioxidant effects of vitamin D on diabetic mice [21]. Vitamin D_3_ reduced the damaged tube formation activity of HUVECs induced by cooking oil fume-derived particulate matter [20]. Thus, the effects of vitamin D on endothelial function under high glucose and PM stimulation are worth evaluating. In the present study, the active form of 1,25-dihydroxyvitamin D [1,25(OH)_2_D_3_] significantly reduced mitochondrial ROS production, mitophagy and inflammation following high glucose and PM stimulation, while the circulating form of 25-hydroxyvitamin D [25(OH)D_3_] did not exert a protective effect. NAC and MitoTEMPO are antioxidants specifically targeting cells and mitochondria, respectively, and reduce endothelial damage. Pretreatment with 1,25(OH)_2_D_3_ reduced PM-induced cell damage by inhibiting oxidative stress in HUVECs stimulated with high glucose. However, following high glucose and PM treatment, 25(OH)D_3_ (5 μM) had no effect on reversing the decreased cell viability or reducing mitochondrial ROS levels. Based on these results, only activated vitamin D elicited cytoprotective effects in this study. In addition, some studies suggested that other antioxidants, such as vitamin C, vitamin E, NAC and deferoxamine, elicited protective effects on injury induced by coal dust-derived PM [50, 51]. Vitamin C and vitamin E also protect against other air pollutants, such as ozone and sulfur dioxide [52–54]. We aimed to fully understand the effect of vitamin D on PM and HG-induced endothelial damage by injecting mice with STZ to establish type 1 diabetes and injecting PM into the trachea to simulate air pollution. This animal model is recognized and widely used [55, 56]. Consistent with the results obtained from the cultured endothelial cells, our data showed that vitamin D reduced apoptosis, ROS production, and inflammation in the aortas of STZ- and PM-treated mice in vivo. In addition, vitamin D reduced the expression of p62, LC3B-II and Bnip3. However, we cannot rule out that the in vivo study of the adverse effects of PM on aortic endothelial cells are derived from serum carriers containing secondary signals that are exposed to PM through the lungs [57]. Nevertheless, the current results pose limitations of PM doses used in in vitro and in vivo studies, which are beyond the real-world exposures and warrant further research in the future. To the best of our knowledge, our study provides the first evidence of the effects of exposure to HG on endothelial damage in mice, and PM exhibited a synergistic interaction with these adverse effects. Vitamin D reversed these changes. Taken together, our data provide new insights that coexposure to PM and HG may contribute to the onset and development of CVDs.

## Conclusion

As shown in the present study, exposure to PM under high glucose conditions exacerbates apoptosis and oxidative stress in endothelial cells. In addition, PM and high glucose significantly increased mitophagy and inflammation through the JNK/p38 pathway, and vitamin D pretreatment effectively alleviated this damage in vitro. Furthermore, vitamin D also protects cells in vivo. The present study highlights the role of vitamin D as a therapeutic intervention in the combination of diabetes mellitus and air pollutants.

## Methods

### Preparation of PM

The standard reference material (SRM 2786, average particle size < 4 μm) was purchased from the National Institute of Standards and Technology (NIST, USA). It was collected from Prague, Czech Republic in 2005. The certificate of analysis of SRM 2786 reveals that PM is composed of selected polycyclic aromatic hydrocarbon components, including polycyclic aromatic hydrocarbons, nitro-substituted PAH, polybrominated diphenyl ether homologs, hexabromocyclododecane isomers, sugars, polychlorinated dibenzo-p-dioxin and dibenzofuran congeners and inorganic components [58]. The stock suspension of SRM 2786 was first dissolved in M199 culture medium (Gibco, MA, USA) at a final concentration of 10 mg/mL, sonicated 3 times for 10 min, frozen and stored at -20 °C. The stock solution used for intratracheal injection was dissolved in PBS (10 mg/mL), sonicated 3 times for 10 min and stored at -20 °C [59]. The solution was then vortexed vigorously for 2 min to disperse the particles before use in each experiment.

### Cell culture and treatment

Human umbilical vascular endothelial cells (HUVECs) were obtained from the American Type Culture Collection (ATCC, MD, USA) and cultured in M199 medium (Gibco) supplemented with 10% fetal bovine serum (FBS, Biological Industries, CT, USA), 17 μg/mL heparin (Sigma, MO, USA), 30 μg/mL ECGS (Millipore, MA, USA), 20 mM HEPES (Bionavas, ON, Canada), 1% sodium pyruvate (Biological Industries), 1% L-glutamine (Biological Industries) and 1% penicillin/streptomycin/amphotericin (Biological Industries) at 37 °C in a humidified incubator with 5% CO_2_. HUVECs from passages 2–5 were used in the experiment. HUVECs were seeded in a 12-well plate at 80% confluence, unless indicated otherwise. In the subsequent experimental procedure, the medium was removed and replaced with low-serum medium (M199 containing the aforementioned substances except for 2% FBS) for 24 h. Cells were pretreated with 30 mM glucose for 24 h and then treated with 50 μg/mL PM for 8 h to simulate hyperglycemia and air pollution and to determine whether PM may exhibit increased toxicity in a hyperglycemic environment that simulates the conditions observed in patients with diabetes.

Before exposure to PM, HUVECs were pretreated with circulating vitamin D [25(OH)D_3_, Cayman, MI, USA], active vitamin D [1,25(OH)_2_D_3_, Cayman] or Mito-TEMPO (Santa Cruz, TX, USA) for 12 h or N-acetylcysteine (NAC, Sigma) for 1 h. HUVECs were randomly divided into the following treatment groups: (1) control group (normal glucose, 5.5 mM); (2) PM group (50 μg/mL); (3) high glucose group (HG, 30 mM); (4) HG + PM group; (5) HG + PM + 1,25(OH)_2_D_3_ (50 or 100 nM) group; (6) HG + PM + 25(OH)D_3_ (5 μM) group; (7) HG + PM + NAC (10 mM) group and (8) HG + PM + Mito-TEMPO (500 nM) group.

### Cell viability assay

The 3-(4,5-dimethylthiazol-2-yl)-2,5-diphenyltetrazolium bromide (MTT) assay (Sigma) was used to determine the cytotoxicity of PM and high glucose toward HUVECs. HUVECs (1.5 × 10^4^ cells/well) were seeded in 96-well plates. After the administration of different treatments, MTT reagent (0.5 mg/mL in M199 medium) was added to the cells and incubated for 2 h at 37 °C in the dark. Thereafter, the medium was aspirated and 50 μL of dimethyl sulfoxide (DMSO) was added to dissolve the formazan and incubated at 37 °C for 15 min. A microplate reader (Biotek, VT, USA) was used to measure the absorbance at 550 nm. Cell viability (%) was calculated as (A550 of treated cells/A550 of related control) × 100%.

### Annexin V/PI analysis

According to the manufacturer’s protocol (BioLegend, CA, USA), the FITC-Annexin V Apoptosis Detection Kit with PI was used to determine cell death in different groups. Briefly, the cell pellet was resuspended in 100 μL of binding buffer and then incubated with 2.5 μL of Annexin V-FITC and 5 μL of PI on ice for 20 min in the dark. Finally, 250 μL of binding buffer were added to stop the reaction and the samples were analyzed with a FACSCalibur flow cytometer (BD, CA, USA). The cell population was subdivided into viable cells (Q4 quadrant, Annexin V^−^/PI^−^), early apoptotic cells (Q3 quadrant, Annexin V^+^/PI^−^), late apoptotic cells (Q2 quadrant, Annexin V^+^/PI^+^), and necrotic cells (Q1 quadrant, Annexin V^−^/PI^+^). Apoptotic cells are composed of early and late apoptotic cell populations (Annexin V^+^/PI^−^ and Annexin V^+^/PI^+^).

### TUNEL assay

HUVECs were seeded in a 12-well plate preloaded with coverslips at 80% confluence. According to the manufacturer’s instructions (in situ cell death detection kit, Roche, CA, USA), apoptosis was detected using a fluorescent TdT-mediated dUTP nick end labeling (TUNEL) detection kit. In addition, 4',6-diamidino-2-phenylindole (DAPI) (Southern Biotech, AL, USA) was subsequently used for nuclear staining. The percentage of TUNEL-positive nuclei relative to total nuclei was blindly determined in five 40 × fields of view that were randomly selected from each coverslip on each slide.

### Analysis of mitochondrial ROS and cellular ROS levels

MitoSOX Red (Invitrogen, MA, USA) was used to measure mitochondrial ROS production. HUVECs were incubated with medium containing 5 μM MitoSOX Red for 10 min at 37 °C. Red fluorescence was measured at 580 nm after excitation at 510 nm using a fluorescence microscope (Leica, Wetzlar, Germany) or an LSRFortessa flow cytometer (BD). Intracellular ROS include superoxide anions and oxygen free radicals. The level of superoxide anions in the cells was measured using dihydroethidium (DHE) (Invitrogen). HUVECs were incubated with medium containing 5 μM DHE at 37 °C for 15 min. DHE is converted into ethidium using superoxide anions, showing red fluorescence, which is measured using fluorescence microscopy and flow cytometry. The production of intracellular oxygen free radicals was monitored by treating the cells with 2', 7'‐dichlorodihydrofluorescein diacetate (H_2_DCF-DA; Invitrogen). Nonfluorescent H_2_DCF-DA quickly diffuses through the cell membrane and is hydrolyzed by intracellular esterase into a form that is sensitive to oxidation, namely, dichlorodihydrofluorescein (H_2_DCF). This form serves as a substrate for an oxidant in the cell to produce highly fluorescent dichlorofluorescein (DCF). The measured DCF fluorescence intensity is proportional to intracellular oxygen free radicals. HUVECs were incubated with media containing 10 μM H_2_DCF-DA at 37 °C for 15 min. H_2_DCF-DA is converted into DCF by oxygen free radicals, showing green fluorescence, as observed using fluorescence microscopy and flow cytometry. Images were captured using a fluorescence microscope and quantified using flow cytometry.

### Measurement of the mitochondrial membrane potential

MitoStatus TMRE (BD) and JC-1 (Cayman) staining were used to evaluate the changes in mitochondrial membrane potential (Δψm). HUVECs were incubated with media containing 100 nM MitoStatus TMRE at 37 °C for 15 min. Δψm was then analyzed using an LSRFortessa flow cytometer. In addition, cells were incubated with media containing 1 μg/mL JC-1 at 37 °C in the dark for 30 min. The green fluorescence reflects the monomer of JC-1, while the red fluorescence represents the aggregated form. The images were then captured using a fluorescence microscope and analyzed using an LSRFortessa flow cytometer.

### ATP measurement

HUVECs received the treatment described in the experiment. HUVECs were isolated by trypsinization and resuspended in RIPA buffer on ice. The sample was centrifuged at 14,000 g for 20 min at 4 °C, and the supernatant was further analyzed using the ATP Determination Kit according to the manufacturer’s protocol (Thermo, MA, USA). Then the ATP level was reported as the fold change compared to the control group.

### Western blot analysis

Western blot analysis was performed as described in our previous study [60]. Homogenized aortic tissues and cells were lysed in RIPA buffer [50 mM Tris, pH 7.4, 150 mM NaCl, 1% NP-40, 0.5% sodium deoxycholate, and 0.1% sodium dodecyl sulfate (SDS)]. Equal amounts of protein samples (30 μg) were electrophoresed on SDS–polyacrylamide gels and transferred onto polyvinylidene fluoride (PVDF) membranes (Millipore). The nonspecific protein binding was blocked by incubating the membrane with 5% milk in 1 X TBST (10 mM Tris/150 mM NaCl/0.05% Tween‐20, pH 7.5) for 1 h at room temperature (RT). These membranes were incubated with the following primary antibodies: cytochrome c (1:1000, 4280S, Cell Signaling, MA, USA), PUMA (1:2000, ab9643, Abcam, MA, USA), SOD1 (1:2000, GTX100554, Genetex, CA, USA), SOD2 (1:2000, GTX630559, Genetex), LC3B (1:2000, 2775S, Cell Signaling), SQSTM1/p62 (1:2000, 5114S, Cell Signaling), Bnip3 (1:1000, 44060S, Cell Signaling), Bnip3 (rodent specific, 1:1000, 3769S, Cell Signaling), phospho-ERK (1:2000, sc-7383, Santa Cruz), phospho-SAPK/JNK (1:2000, 9251S, Cell Signaling), phospho-p38 MAP kinase (1:2000, 9211S, Cell Signaling), ICAM-1 (1:1000, sc-8439, Santa Cruz), VCAM-1 (1:2000, ab134047, Abcam), and GAPDH (1:5000, 60,004-1-Ig, Proteintech, IL, USA) at 4 °C overnight. Then they were incubated with horseradish peroxidase-conjugated goat anti-mouse or rabbit IgG (1:5000, 115-035-003 or 111-035-144, Jackson ImmunoResearch, MA, USA). Luminata™ Crescendo ECL (Millipore) was used to detect the immunoreactive bands, and images were captured using a Biospectrum Imaging System (UVP, CA, USA). The optical density analysis of Western blots was analyzed using ImageJ software (NIH, USA). The relative protein levels were calculated and normalized to the internal control GAPDH protein.

### BNIP3 knockdown

HUVECs were seeded in a 12-well plate at a confluence of 70%. Then, HUVECs in each well were transfected with 25 nM SMARTPool siRNA targeting Bnip3 (siBnip3) or scrambled siRNA (siScr) (Dharmacon, CO, USA) according to the instructions provided with Lipofectamine 3000 (Thermo). On the second day, HUVECs were shifted to low-serum M199 medium. After 24 h, HUVECs were incubated with high glucose (30 mM) M199 medium for another 24 h. Finally, HUVECs were exposed to PM (50 μg/ml) for 8 h. HUVECs were harvested for further analysis.

### Acridine orange (AO) staining

AO (Invitrogen) is a cell-permeable dye that targets acidic compartments such as lysosomes and autolysosomes. The orange fluorescence produced by the aggregation of AO combined with lysosomes or acidic cell vesicles is suitable for observing autophagy. Cells exposed to different treatments were washed with PBS and incubated with medium containing AO (1 μg/mL) at 37 °C for 5 min. The cells were gently washed with PBS and checked under a fluorescence microscope (Leica).

### RFP-GFP-LC3B assay

Premo™ Autophagy Tandem Sensor RFP-GFP-LC3B (Thermo) was used to evaluate the number of autophagosomes and autolysosomes and analyze autophagy flux. Briefly, HUVECs were grown on coverslips in a 24-well plate, HUVECs were infected with insect baculovirus with mammalian promoter carrying expression cassettes that encode tandem fluorescence-tagged LC3B, in which GFP is more sensitive to acidic conditions (autolysosome) than RFP. After 24 h of transduction, HUVECs were treated as described above. HUVECs were fixed by 4% formalin for 10 min at RT, followed by nuclei staining with DAPI for 5 min at RT. The images were examined and captured under a fluorescence microscope. Ten cells per treatment were used for quantification.

### Transmission electron microscopy

HUVECs were prefixed with 2% paraformaldehyde and 2% glutaraldehyde fixative at 4 °C overnight, then postfixed with 1% osmium tetroxide (OsO_4_), dehydrated in a series of alcohol solutions, and then embedded in Epon. Ultrathin sections on grids were stained with uranyl acetate and lead citrate. The samples were observed with an H-7500 transmission electron microscope (Hitachi, Japan). Images were captured using AMT Image Capture Engine V5.4.2 (Advanced Microscopy Techniques, MA, USA).

### ELISA (enzyme-linked immunosorbent assay)

According to the manufacturer’s protocol, the human IL-6 ELISA MAXTM Deluxe Set (BioLegend, CA, USA) was used to measure the IL-6 level in the cell culture supernatant. A microplate reader was used to measure the absorbance at 450 nm. The data are reported in pg/mL protein and are presented as the means ± standard errors of the means (S.E.M.) of 5 independent samples.

### Monocyte adhesion assay

HUVECs received the treatment indicated in each experiment as described above. THP-1 cells, which were originally derived from human acute monocytic leukemia and obtained from ATCC, were labeled with 10 mM BCECF/AM (Thermo) for 1 h at 37 °C. Labeled THP-1 cells were washed and incubated with HUVECs for 1 h at 37 °C. The medium containing the unattached THP-1 cells was removed and the remaining cells were washed twice with PBS. In each experiment, images of THP-1 cells adhering to HUVECs were captured using a FLoid™ Cell Imaging Station (Thermo), and the number was counted in three randomly selected images.

### Animal model

Six-week-old male C57BL/6 mice weighing 20 g were obtained from the Laboratory Animal Center of National Taiwan University. Mice were housed on a 12-h light/dark cycle and water and chow diets were provided ad libitum. The mice were randomly divided into the following treatment groups (N = 7): (1) control group; (2) PM exposure group (PM); (3) diabetes group (STZ); (4) STZ + PM group and (5) STZ + PM + 1,25(OH)_2_D_3_ group.

Moderate diabetes was induced by administering an intraperitoneal injection of streptozotocin (STZ, Sigma) at a dose of 55 mg/kg (in 0.1 M sodium citrate buffer, pH 4.5) for five consecutive days after an overnight fast. The mice in the control and PM groups received intraperitoneal injections of sodium citrate buffer as a vehicle treatment. Blood samples were collected from the submandibular region. Mice with fasting blood glucose levels higher than 280 mg/dL were considered to have diabetes. The mean blood glucose concentration in STZ-induced mice was 607.73 ± 55.70 mg/dL, similar to in vitro cells treated with high glucose concentration (30 mM, 540 mg/dL).

Vitamin D (1,25(OH)_2_D_3_) was intraperitoneally injected at a dose of 7 μg/kg daily for two weeks. Vitamin D has low mammalian toxicity as it is classified as a Class III toxic chemical. Oral LD_50_ of vitamin D in mice is 42.5 mg/kg [61]. As shown in Additional file 1: Fig. 3, liver and kidney function assessed by ALT and serum creatinine showed that the dose of vitamin D treatment in vivo did not cause adverse effects in animals*.* On the second and eighth days of vitamin D treatment, the mice were injected with PM (10 mg/kg, dissolved in PBS) into the trachea under anesthesia to simulate air pollution. The mice were anesthetized with 2% isoflurane inhalation, the trachea was exposed, and then an insulin syringe was utilized to puncture the anterior wall of the trachea at a 45° angle to avoid impaling the posterior wall. The neck and trachea were sutured after the PM injection and the mice were returned to their home cage after revival. Mice in the control and STZ groups received intratracheal injections of PBS as a vehicle treatment. No mice died from the intratracheal injection process and all mice survived until the end of the experimental process. At the end of the treatment, the aorta and serum were collected, and the serum alanine aminotransferase (ALT), creatinine, and blood glucose levels were measured. Part of the aortic tissue was fixed with 4% buffered paraformaldehyde and embedded in paraffin for immunohistochemical and hematoxylin–eosin staining. The remaining part was quickly frozen in liquid nitrogen for protein extraction to examine the levels of ICAM-1, VCAM-1, p62, LC3B, and Bnip3 using Western blotting. Briefly, aortic tissue was lysed in lysis buffer supplemented with phosphatase and protease inhibitors. The lysate was then centrifuged at 14,500 × g at 4 °C for 20 min. The supernatant was stored at − 80 °C until further study.

### Immunohistochemistry

Five micrometer-thick sections were cut from the paraffin blocks. The sections were deparaffinized in an oven at 60 °C for 1 h and then gradually rehydrated with 100%, 95%, 85%, and 75% graded alcohol solutions for 5 min each. After antigen retrieval using 10 mM sodium citrate, the endogenous peroxidases were inactivated with 3% hydrogen peroxide for 10 min at RT. Sections were double-stained with ICAM-1 (1:200 dilution) and CD31 (a marker for endothelial cells, 1:100, Abcam) antibodies to determine whether ICAM-1 was expressed in endothelial cells. After washes with PBS, the sections were incubated with an Alexa Fluor 488-conjugated secondary antibody (1:200 dilution) to detect ICAM-1 and Alexa Fluor 594-conjugated secondary antibody (1:100 dilution) to detect CD31. Subsequently, the slides were counterstained with a DAPI solution and examined using fluorescence microscopy.

### Measurement of ROS production in the aorta

For DHE staining, tissues were placed in OCT (Fisher Scientific, MA, USA) and then snap frozen in liquid nitrogen. Then, 7 μm frozen sections were prepared using a cryostat (Leica Biosystems, IL, USA). Tissues were rinsed with ddH_2_O for 5 min, and then incubated with 10 μM DHE at 37 °C for 10 min. Slides were washed with PBS, mounted with mounting medium and observed under a fluorescence microscope.

### Statistical analysis

The experimental group for in vitro studies includes independent biological replicates, with the number of repetitions ranging from 5–8 times. Seven animals per group were studied in vivo. Data are presented as the means ± S.E.M. The statistical analysis was performed using SPSS software (version 17.0; IBM, NY, USA). One-way analysis of variance followed by Dunnett's post hoc test was used for multiple comparisons. Differences between 2 groups were calculated using the Student’s t-test. *P* < 0.05 was considered statistically significant.

## Supplementary Information

**Additional file 1.** Supplementary Material.

## Data Availability

The datasets used and/or analyzed during the current study are available from the corresponding author upon reasonable request.

## Abbreviations

PM: Particulate matter
HG: High glucose
ROS: Reactive oxygen species
HUVECs: Human umbilical vein endothelial cells
SOD1: Superoxide dismutase 1
SOD2: Superoxide dismutase 2
LC3B: Microtubule-associated protein 1 light chain 3β
Bnip3: BCL2 interacting protein 3
ICAM-1: Intercellular adhesion molecule-1
VCAM-1: Vascular cell adhesion molecule-1
STZ: Streptozotocin
CVD: Cardiovascular disease
ECs: Endothelial cells
