# Supplementary material for: Combined exposure to fine particulate matter and high glucose aggravates endothelial damage by increasing inflammation and mitophagy: the involvement of vitamin D
Source: Part Fibre Toxicol. 2022 Mar 29;19:25. doi: 10.1186/s12989-022-00462-1 (PMC8966234; doi:10.1186/s12989-022-00462-1)
Supplement: Supplementary file 1 — Additional file 1. Supplementary Material. [file 12989_2022_462_MOESM1_ESM.pdf]

Supplementary Material

Combined exposure to fine particulate matter and high glucose aggravates endothelial damage by increasing inflammation and mitophagy: The involvement of vitamin D

Tsai-Chun Lai, Yu-Chen Chen, Hui-Hua Cheng, Tzu-Lin Lee, Jaw-Shiun Tsai, I-Ta Lee, Kuo-Ti Peng, Chiang-Wen Lee, Lee-Fen Hsu, and Yuh-Lien Chen

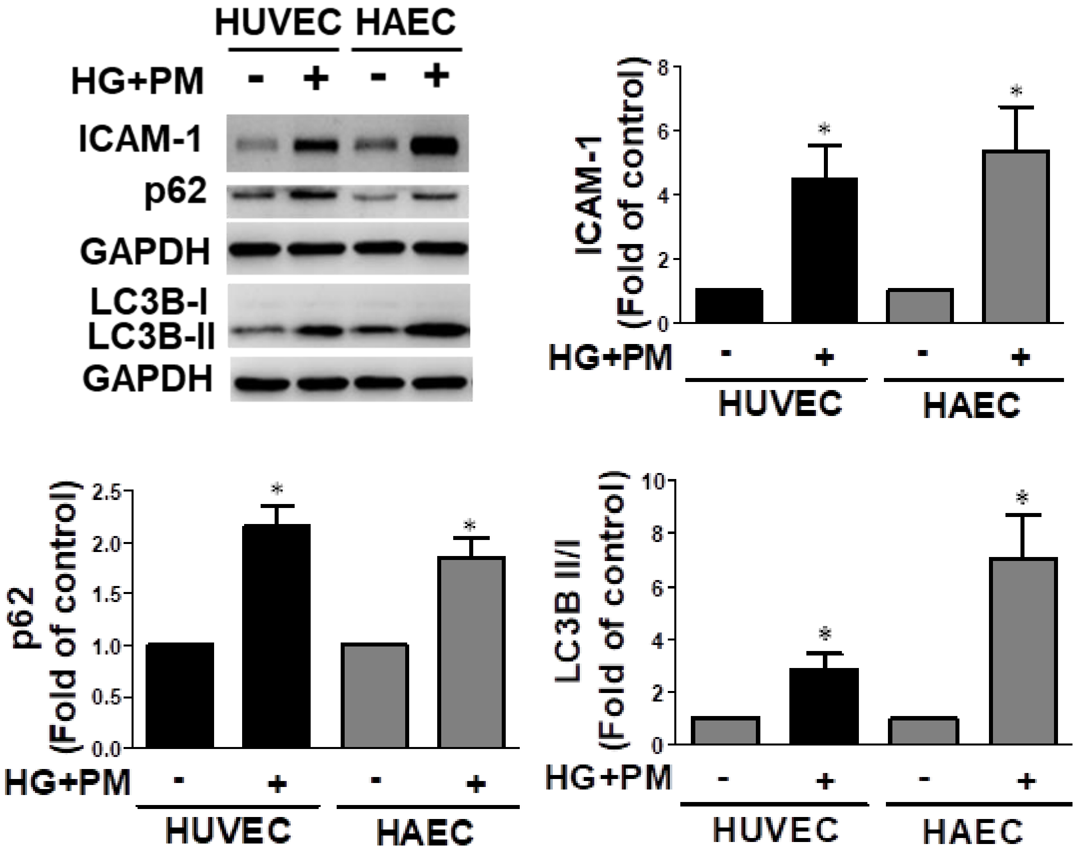

Supplementary Fig. 1 Effects of combined high glucose and PM exposure on ICAM-1, p62 and LC3B expression in HUVECs and HAECs. HUVECs or HAECs were

pretreated with high glucose (30 mM) for 24 h and then treated with PM (50 μg/mL) for 8 h. The levels of ICAM-1, p62 and LC3B expression were detected using Western blotting. N=5. *P<0.05 compared with the control group.

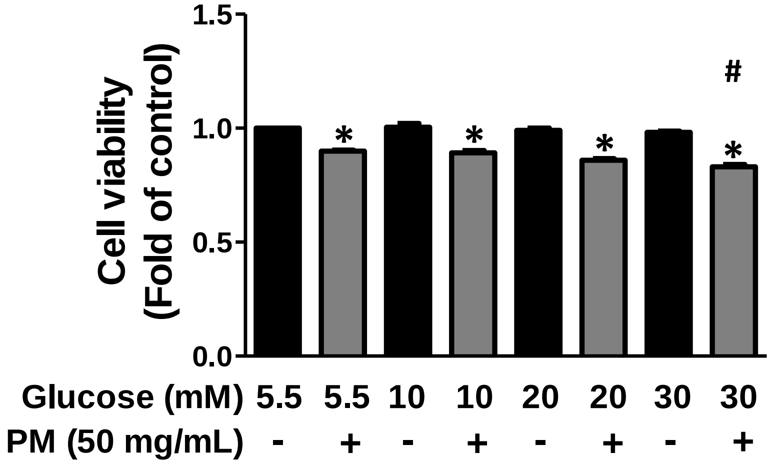

Supplementary Fig. 2 The effect of glucose concentration on the viability of PM-treated HUVECs. HUVECs were pretreated with 5.5 mM, 10 mM, 20 mM, and 30 mM glucose for 24 h and then treated with PM (50 μg/mL) for 8 h. Cell viability was assessed using the MTT assay. N=5. *P<0.05 compared with the 5.5 mM, 10 mM, 20 mM, or 30 mM glucose group; †P<0.05 compared with the 5.5 mM glucose+PM group or the 10 mM glucose+PM group; $^{\#}$P<0.05 compared with the 20 mM glucose+PM group.

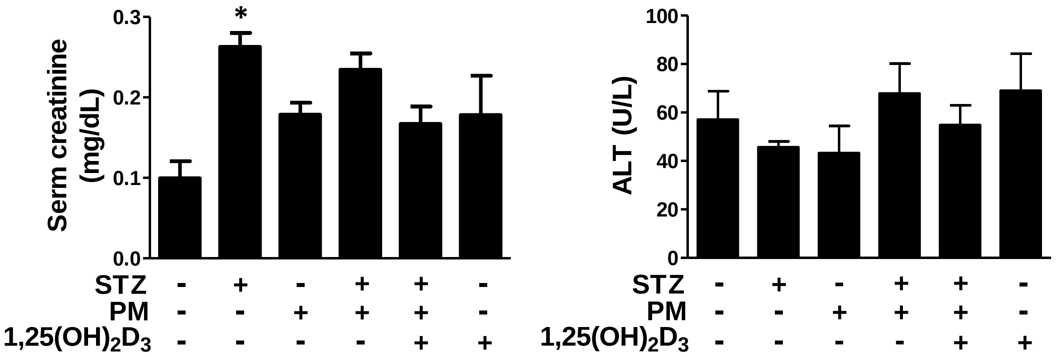

Supplementary Fig. 3 Biochemical analysis of serum creatinine and ALT. Diabetes was induced in mice by intraperitoneally injecting STZ (55 mg/kg). Mice received PM (10 mg/kg) via intratracheal injection under anesthesia to simulate exposure to air pollution. 1,25$(OH)_2D_3$ was administered at a dose of 7 μg/kg daily for two weeks. The levels of serum creatinine and ALT were analyzed. N=7. *$P<0.05$ compared with the control group.
